# On the Value of a Large Supply of Food in Nervous Diseases

**Published:** 1870-11

**Authors:** 


### *On the Value of a Large Supply of Food in Nervous Disorders.*

Among the various therapeutical agents and innumerable drugs advocated and employed for the relief of nervous weakness, and the cure of the disorders which thence arise, it is possible that the unaided effects of food may not in all cases have met with the trial they deserve. Patients thus affected are told to live well and adopt a generous diet, but the generosity of this is usually estimated by the amount of port wine or other alcoholic stimulant, rather than by that of the bread, mutton, or beef.

Certain chronic invalids who have been brought under my notice, have been lifted out of their former condition of "nervousness" by a large increase in the quantity of their food. They have been people suffering from some general neurosis, taking the form of an insanity of a low and depressed character, or hypochondriasis, hysteria, alcoholism, or neuralgia, affections closely allied one to another, which may be witnessed, in one form or another, in individuals inheriting the same neurotic temperament. We may see different members of the same family displaying, one insanity, another neuralgia, a third hypochondriasis, while the conversion of one variety to another is a matter of every-day observation.

A paper on "Indiscriminate Stimulation in Chronic Disease," from Dr. Anstie's pen, appeared in this journal in July last. With all that he says I cordially agree, and more on this portion of the subject need not be urged at present. It is a matter of the gravest importance that the treatment of such cases should not be conducted by means of unlimited supplies of alcohol.

If we inquire into the past history of nervous patients, and have the opportunity of learning accurately the facts thereof, we often find that for a considerable time the supply of daily food has been in no degree adequate to the necessities of the individual. Here is a common case. A man somewhat past middle life, but whose years do not imply senile decay, becomes unfit for business, fidgety, irritable, depressed, or even melancholic to the extent of insanity. We hear that he has been a hard-working man of business, always nervous, and very probably an indifferent sleeper. Being most heavy for sleep in the morning, he has risen at the latest moment, and, snatching a mouthful of breakfast, has hurried off to catch the train or omnibus, worried and anxious lest he fail to reach his office at the hour appointed. At lunch-time, if he be really hard-worked, he takes, not a meal, but a sandwich or biscuit, eaten perhaps standing, and often bolted in so great a hurry that digestion is difficult; he tells us that he dare not take more of a meal in the middle of the day, for he would be rendered unfit for the remainder of his work. In the evening, with what appetite he may, he eats his dinner, perhaps not before half past seven o'clock. Now, granting that his dinner is amply sufficient, such a man lives on one meal a day with very little besides. These are the persons who cannot go on without frequent holidays; nervous by inheritance, they break down because they are insufficiently fed. A holiday, during which they live better, builds them up again for a time, again to break down; often to fall into the condition above mentioned. Another class among whom we may frequently witness the same result and corresponding symptoms, are the clergymen, who, for various reasons, deny themselves an adequate amount of food. Either they fast rigidly, according to the rule and doctrine of the day, often allowing some hours to elapse before they break their fast, or they think that hearty eating is a snare and a carnal enjoyment, or they hold it sinful to eat their fill while others are in want. Whatever the cause, certain it is that many of the clergy break down in one or the other of the forms of nervous disorder already enumerated, and an enlarged dietary is to them a necessity. A vast number of women, for one reason or another, take a very small supply of food: some think it unladylike to eat heartily; some eat on the sly, and when this is not practicable, go without. Many from the lives they lead are doubtless correct in saying they cannot eat because they have no appetite. These stay in the house from month to month, or never venture beyond the door except in a carriage, because ladies do not walk in the streets. Others have misgivings on the score of their digestion. Like many women who lead sedentary lives, and habituate themselves to passing long periods without action of the bowels, they suffer greatly from constipation, which is looked upon as an indication and a warning that they ought not to eat. So they starve themselves, and fancy that if they abstain from food it is of little consequence whether they pass a motion once a week or once a fortnight.

It may be well to consider somewhat more in detail the various neuroses which have been mentioned.

The first on the list is low nervous depression, commonly known as melancholia, the most formidable of all that have been named, the one most likely to run in a short time into serious and even fatal insanity, but which, if arrested at an early stage, is often singularly amenable to treatment. In almost every example of this variety, and almost from the commencement, we find a marked disinclination to take food, and in extreme cases it can only be administered by some kind of forcible feeding. In milder cases, and at an early period, it will be taken if we insist upon it, and the result of a large supply is frequently manifested in a very brief time. It has been frequently asserted by many writers, that refusal of food on the part of melancholic patients is due to dyspepsia, and in confirmation of this opinion they point to the foul and furred tongue, the obstinate constipation, and the fœtor of breath so constantly observed in such patients; but this condition of the tongue and fœtor are due, I am convinced, not to gastric disturbance, but to the generally depressed and devitalized state of the individual; and the best proof of the absence of dyspepsia is that, although we suddenly compel the ingestion of what, compared with that previously taken, may be called an enormous quantity of nourishment, the stomach by no means rejects it, but, on the contrary, retains and digests it, as is shown by the rapid amelioration which takes place. It is inconceivable that dyspepsia can be the cause of refusal of food when the administration of it is unattended by sickness or inconvenience, even when that which is taken into the stomach is not light invalid-diet, but such substance as beef or mutton. From my own observations, and from the subsequent confession of patients, I am inclined to believe that the refusal of food is in almost every case the result of delusion, this being in turn the result or interpretation in consciousness of the extreme nervous depression and exhaustion under which they are laboring. They are too wicked to live, too wicked to eat; it is sinful to pamper their flesh and their appetites; they beg for cold water and dry bread, but the idea of a good dinner their soul abhors. If we see such sufferers at an early stage when forcible feeding is not necessary, and they will take that which is ordered, merely protesting against the uselessness or wickedness of the proceeding, we may prescribe a very large amount of food without fear, nay, with a constant expectation of the greatest benefit. What the food is to consist of is a point on which little need be said. It is not necessary to adhere to a sick diet,—to beef-tea or boiled mutton, to essences of beef or Liebig's food or any other of the concentrations so loudly recommended. The ordinary diet-list of the individual in health may be given without hesitation—fish, game, poultry, meat, puddings, and the rest. His appetite should be stimulated by variety, and his dishes may be savory as well as wholesome; but the supply must be large. Such patients for the most part have accustomed themselves to eat during the day a scanty and insufficient amount, and we shall be told that latterly they have not taken half their usual quantity. It is not too much to say that they require double that which they have so long taken; and as we shall not be able to induce them to eat double the quantity at a single meal, it will be necessary to multiply the number of the meals. Instead of breakfast, lunch, and dinner, two of which have probably been but the semblance of a meal, we may institute a series of feedings after this kind: First, something may be given early in the morning, before the patient gets up, as rum and milk, egg and milk, chocolate or *cafe au lait.* This will be useful in allaying the feeling of extreme depression and dispelling the gloom and suicidal thoughts so constantly present on first waking. Next, breakfast may be taken, after dressing, and between it and two o'clock lunch something else, as beef-tea or a sandwich. The dinner hour should not be later than six, and at bedtime some light kind of supper should not be omitted. By this kind of division, food may be administered six times in the day; and if the patient wakes in the night, and is restless and nervous, and disinclined to sleep again, food, taken even in small quantity, will often bring back sleep. With all the food may be given a reasonable amount of wine, or wine and stout, and this not by way of curing the disorder by stimulants, but because in conjunction with them less food appears to be required, and also because the addition of some wine or beer often renders the taking of the food more easy to the patient.

Now the latter, and it may be the friends, will protest loudly that it is impossible to take this quantity; he will assign every conceivable reason for avoiding it; but if we are firm and insist, and, if necessary, cause him to be fed with a spoon, he will retain and thrive on it, and in a few weeks, or even days, will show very marked signs of its good effect. Patients have recovered under this treatment in a singularly rapid manner. Some learn in a short time to appreciate the benefit of the food, and miss their meal if from any cause they are unable to take it at the appointed hour, and some have gone on for years after their recovery, taking, not the quantity prescribed during the acute stage of their illness, but one very much larger than that on which they had endeavored to live for so long, and under such a change of regimen have lost all trace of the depression and hypochondria from which they formerly suffered. Although beef-tea, chocolate and milk have been mentioned as articles of diet, it by no means follows that liquids are to predominate; on the contrary, solid food is far better as sedative, and also far more nutritious, and it may be taken as in health. Much has been said concerning the advantage of fatty food in nervous disorders, and sugar has been thought to disagree with these patients; but in my own experience I have found that all the various foods—the fatty, the starchy, sugar and meat—may be given in due proportion at any rate to the individuals now under consideration. If this amount and description of diet be administered, there will be little need of medicine, except perhaps of Morphia or Chloral to procure sleep at the commencement of the treatment.

The next variety of neurosis in which the efficacy of abundant food is markedly shown, is alcoholism, whether acute or chronic. I shall not here enter upon the question whether delirium tremens is ever caused by the removal of alcohol; controversy upon this point is not yet at an end, and it will exist so long as we are ignorant of the precise pathological cause and condition of delirium. But whether alcohol is to be entirely avoided or not in the treatment of this disease, it is, I believe, an established fact that abundant nourishment, not spoon diet, but solid food, should be given as soon as the stomach can retain it. The irritability of the latter is a difficulty to be met in various ways, and owing to this we may at first be obliged to resort to concentrations of food—Liebig's extract, various preparations of beef-tea, and so on. It would appear that sleep is far more easily procured, and that medicines given for it are far more efficacious, if an abundant supply of nourishment is administered at the same time.

It is rather, however, in chronic alcoholism that the good effect of food may be witnessed. Here it is often of the greatest consequence to abolish alcoholic stimulants entirely; in fact, in such abolition lies the only hope of effecting the reformation of the chronic drinker. The intense sinking and craving for the accustomed stimulants may often be effectually met by food, especially if a small quantity be given frequently, as recommended already. Such patients are unquestionably most difficult to deal with; they assign reasons of all kinds for rejecting food, and for being treated by their favorite remedy. They are faint, they require support, they suffer from stomach ailment, from pain, from want of appetite, nausea, or sinking; but they rarely vomit that which they take if drink is withheld, and this is a tolerably sure sign that the stomach is equal to the digestion of the food. The symptoms of alcoholism need not be here described; but whether they be the transient and immediate results of a heavy debauch, or the grave signs of commencing degenerative change of the nerve tissues, which runs to alcoholic paralysis, epilepsy, or dementia, food is equally demanded, and is in fact the one thing which can arrest this degeneration by supplying nutritive elements in large quantities. The recovery in such cases is often astonishing. I lately saw a young man who for many weeks was completely paraplegic, but who nevertheless entirely regained the use of his limbs. The recoveries, too, from alcoholic dementia are often equally surprising; in fact, there seems scarcely any state from which recovery may not take place if the disease has not existed for a long period, and if we are able to withdraw all alcohol, and administer nourishment in large quantity.

There are a number of people whose nervous temperament displays itself in symptoms which are called, in common parlance, hysterical, or hypochondriacal. While young they are termed hysterical, especially if they are women; when older, they are known as hypochondriacs, and their nervousness then takes, for the most part, the form of depression and anxiety, or even suffering on account of some fancied bodily disorder. Now, although hysteria is held by some to be peculiar to women, and discussions are raised as to whether the seat of it is in the womb or the ovaries, or elsewhere, it is, I think, a fact that there is the closest connection between these two neuroses; that the condition of my patients would be as well described by the one term as by the other, and that the subjects of both the one and the other may be of either sex.

Few of these will be found to take an adequate supply of proper food, and those who take the least will present the most distressing symptoms of their disorder. The hypochondriacal direct their attention to the digestive organs more frequently than any other region. They suffer from constipation, flatulence, and a host of other evils, and for this reason either shun food, or eat most unwholesome and extraordinary combinations irregularly or at long intervals. Hysterical women—I am now speaking of young girls—are especially prone to eat irregularly; to take food, if possible, when unnoticed; to eat altogether a very inadequate quantity, and to eke it out by an inordinate proportion of stimulants. If we look at such, especially hypochondriacal, their whole aspect betokens innutrition. Often they are miserably thin; if they are given to drink they may be fat, but their flabby tissues speak of low organization and defective power. It is evident that the nervous energy of such people is very low; this is manifested by their mental depression and disturbance, and the defect must be supplied from some quarter or other. But whence can a supply of force come except from the material of food taken into the system by the alimentary organs? Moral measures are, it is said, and said truly, essential to the recovery of such persons. But moral measures constantly fail, because the bodily health does not allow of mental improvement, and is not *pari passu* attended to. As in more marked mental aberration no amount of argument, proof, or moral suasion will expel a delusion which vanishes of itself when bodily health is renovated; so change of scene, change of persons, and moral treatment of every kind, will fail with the hysterical or hypochondriacal so long as they try to live upon physic or alcohol, or upon a diet almost devoid of nutritive elements.

It may be objected, that some hypochondriacal patients eat, not scantily, but enormously, taking more than is necessary for a person in health. Such are to be found, but in my experience they are the least to be pitied of their class. Though nervous about themselves, and prone to take notice of the slightest indication of anything they may think an ailment, they are not generally depressed or unhappy, but after a fashion of their own, they exert themselves and enjoy life. Such people, I believe, take this amount of food from a feeling that it is to them a necessity, and thus they keep at bay the graver nervous disorder which perpetually threatens them, and the matter of alcoholic stimulants they rarely exceed. Food is to them a stimulus, and were it withdrawn they would speedily show signs of more serious mental mischief.

The other subject on which I propose to say something is neuralgia. It is obvious that any observations upon it must be of the widest and most general character, and that no account can be taken of the special forms of this neurosis, or of any pathological changes connected with it. Believing with many others that neuralgia is one manifestation of impaired sensibility, as other neuroses may be displayed in mental symptoms, and in these alone, I think that the radical cure and not the mere alleviation, is to be found in many cases in the supply of a large amount of nutriment to the nervous system. The confessed failure of drugs in the case of neuralgias, and the mere temporary alleviation by such methods as hypodermic injection, inhalation, or a dose of alcohol, point to the necessity of some more general mode of treatment, which shall effect a greater change in the functions of the nervous system. Those whose experience is greater than mine speak highly of the utility of fatty food, of cod-liver oil, cream, butter, and the like. Whatever the form of food specially indicated, it generally will be found that the entire amount requires to be increased, and that the quantity taken for a series of years has been deficient. It may be that the alimentary system of elderly persons will be found incapable of assimilating the requisite amount. On the intractable nature of the neuralgias of the aged, nothing need here be said.

With two remarks I will conclude. First, in all chronic forms of neurosis, alcoholic stimulants in any but the smallest quantity are a hindrance rather than a help—or productive of evil rather than of good. Secondly, in such disorders the fear, so commonly entertained by both doctors and patients, of "overloading the stomach," producing "biliousness," and the like, is in the majority of cases not realized when the plan of administering food in large quantity is tried. Great opposition will be offered by patients, and every kind of evasion attempted. They will swallow bottles of medicine far more willingly than they will eat sufficient meals at regular intervals. To induce them to do this is often a difficult task, and here moral handling is required. If this is judiciously applied to the patient and the patient's friends, some very remarkable results may be attained.—*The Practitioner.*

